# Serum starvation raises turnover of phosphorylated p62/SQSTM1 (Serine 349), reveals expression of proteasome and N-glycanase1 interactive protein RAD23B and sensitizes human synovial fibroblasts to BAY 11-7085-induced cell death

**DOI:** 10.18632/oncotarget.26295

**Published:** 2018-11-09

**Authors:** Biserka Relic, Edith Charlier, Celine Deroyer, Olivier Malaise, Yannick Crine, Sophie Neuville, Philippe Gillet, Dominique de Seny, Michel G. Malaise

**Affiliations:** ^1^ Department of Rheumatology, GIGA Research, University Hospital Sart-Tilman, Liege, Belgium; ^2^ Department of Orthopedic Surgery, University Hospital Sart-Tilman, Liege, Belgium

**Keywords:** p62/SQSTM1 phosphorylation, proteasome, autophagy, serum starvation, RAD23B

## Abstract

Phosphorylation of p62/SQSTM1 (p62) on Serine 349 (P-Ser349 p62) as well as proteasome dysfunction have been shown to activate the cell protective Keap1/Nrf2 pathway. We showed previously that BAY 11-7085-induced human synovial fibroblast cell death includes autophagy and p62 downregulation. In this work, we have studied expression of P-Ser349 p62 in human synovial fibroblasts. Results showed that P-Ser349 p62 was not detected in synovial cell extracts unless cells were cultured in the presence of proteasome inhibitor (MG132). MG132 revealed P-Ser349 p62 turnover, that was further increased by concomitant autophagy inhibition and markedly enhanced in serum starved cells. Starvation sensitized synovial fibroblasts to BAY 11-7085 while MG132 protected both non-starved and starved cells from BAY 11-7085-induced cell death. Lentivirus mediated overexpression of phosphorylation-mimetic p62 mutant S349E markedly protected synovial fibroblasts from BAY 11-7085. Inhibitor of Keap1-P-S349 p62 interaction, K67, had synergistic effect with MG132. Starvation increased p62 molecular weight, that was reversed by serum and bovine serum albumin re-feeding. Furthermore, starvation markedly induced RAD23B. Increased endo-β-N-acetylglucosaminidase (ENGase) turnover was detected in starved synovial fibroblasts. PNGase F treatment produced faster migration p62 form in human synovial tissue extracts but starvation-like p62 form of higher molecular weight in synovial cell extracts. Co-transfection of NGLY1, with p62 or p62 mutants S349A and S349E markedly stabilized p62 expressions in HEK293 cells. Tunicamycin upregulated p62 and protected synovial fibroblasts from BAY 11-7085-induced cell death. These results showed that P-Ser349 p62 has pro-survival role in human synovial fibroblasts and that de-glycosylation events are involved in p62 turnover.

## INTRODUCTION

Ubiquitin-proteasome-system (UPS) and autophagy are two separate but interconnected protein degradation systems [1]. Ubiquitin binding protein p62, Sequestosome 1 (SQSTM1), has been shown to be a bridge between UPS and lysosomal protein degradation [2, 3]. p62, especially when phosphorylated on Serine 349 (P-Ser349 p62), has high affinity to Keap1, and connect autophagy and UPS to Keap1/Nrf2 antioxidant and cell protective pathway [4]. Of interest, murine p62 (A170) has been cloned from macrophages as an oxidative stress-inducible protein [5]. p62 competes with Nrf2 for Keap1 interaction, preventing Nrf2 degradation [6]. Furthermore, Nrf2 upregulates p62 making the positive feedback loop between them [7]. Human P-Ser349 p62 and mice analog phosphorylation on Serine 351 have the main roles in p62-Keap1 interaction [4, 8], respectively. Both p62 and Nrf2 have been shown as cytoprotective [9]. However, deregulated p62 phosphorylation can lead to metabolic reprograming and cancer development [10]. Mutations in C-terminal of p62 are common in Paget's disease of bone patients [11] and recently discovered S349T mutation of p62 links Paget's disease to Keap1/Nfr2 pathway [12]. Very recently, activation of p62-Keap1-Nrf2 axis was shown to suppress collagen-induced arthritis in mice, in which P-Ser349 p62 was recognized as disease suppressive protein [13].

We have shown previously that BAY 11-7085-induced human synovial fibroblast cell death includes autophagy and p62 downregulation [14]. In this work, we have studied the role of P-Ser349 p62 on human synovial fibroblast fate.

## RESULTS

### UPS inhibition reveals P-Ser349 p62 in human synovial fibroblasts: Significantly higher P-Ser349 p62 turnover in serum starved cells

**Figure 1:**
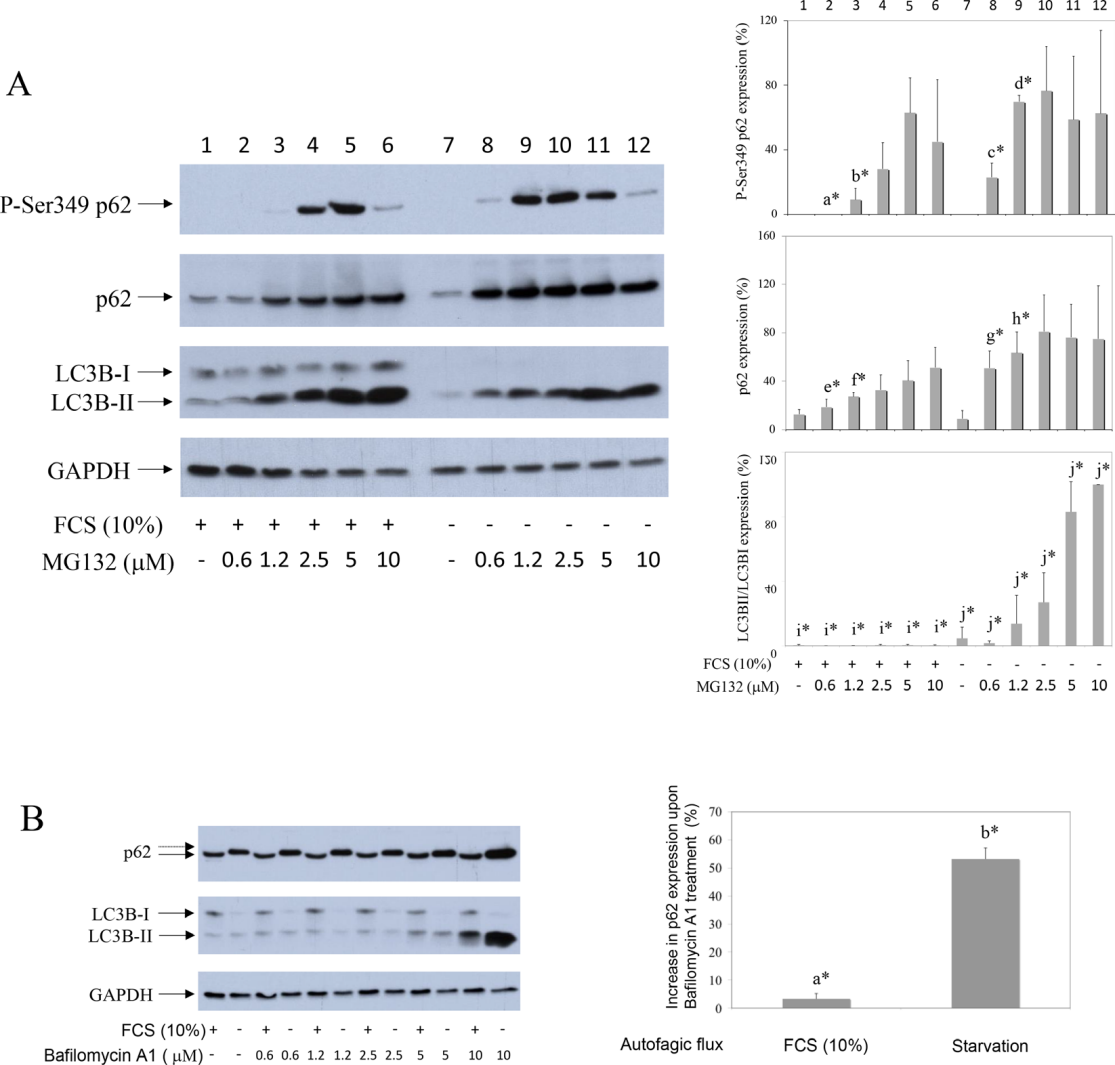
Proteasome inhibitor reveals P-S349 p62 that increases during starvation in human synovial fibroblasts (**A**) Human synovial fibroblasts were treated with different concentrations of proteasome inhibitor MG132 for 24 hours, in the presence or absence of 10% FCS. Western blots show P-S349 p62, p62, LC3B and GAPDH expressions in human synovial fibroblast extracts. Graphics represent average of protein expression from three different experiments done with synovial fibroblasts from three different OA patients. Values were corrected for GAPDH expression and shown as a percentage of maximal protein expression. LC3B is shown as % of LC3BII/LC3BI ratio. a^*^ is statistically lower from c^*^; b^*^ is statistically lower from d^*^; e^*^ is statistically lower from g^*^; f^*^ is statistically lower from h^*^; i^*^ is statistically lower from j^*^. (**B**) Autophagic flux were determined by Bafilomycin A1 treatment of synovial fibroblast for 24 hours, at the indicated concentrations. Graphics represent % of autophagic flux calculated as increase of p62 expression in Bafilomycin (10 μM) treated synovial fibroblasts obtained from three different OA patients. a^*^ is statistically different from control cells and significantly lower than b^*^.

P-Ser349 p62 was not detected in serum cultured (Figure 1A, line 1) or in serum starved human synovial fibroblast extracts (Figure 1A, line 7). However, P-Ser349 p62 accumulated in cells cultured in the presence of proteasome inhibitor MG132 (Figure 1A, lines 3–6 and 8–12). Different concentration of MG132 revealed that serum deprivation, a known inducer of autophagy and LC3B-II (Figure 1B) [15], significantly increased P-Ser349 p62 as well as p62 stabilization (Figure 1A, lines 2, 3 in comparison to lines 8, 9, respectively). These results showed that P-S349 p62 is quickly degraded by proteasome in human synovial fibroblasts, especially during serum starvation.

### Autophagy inhibition further increases MG132-stabilized P-Ser349 p62 in synovial fibroblasts

**Figure 2:**
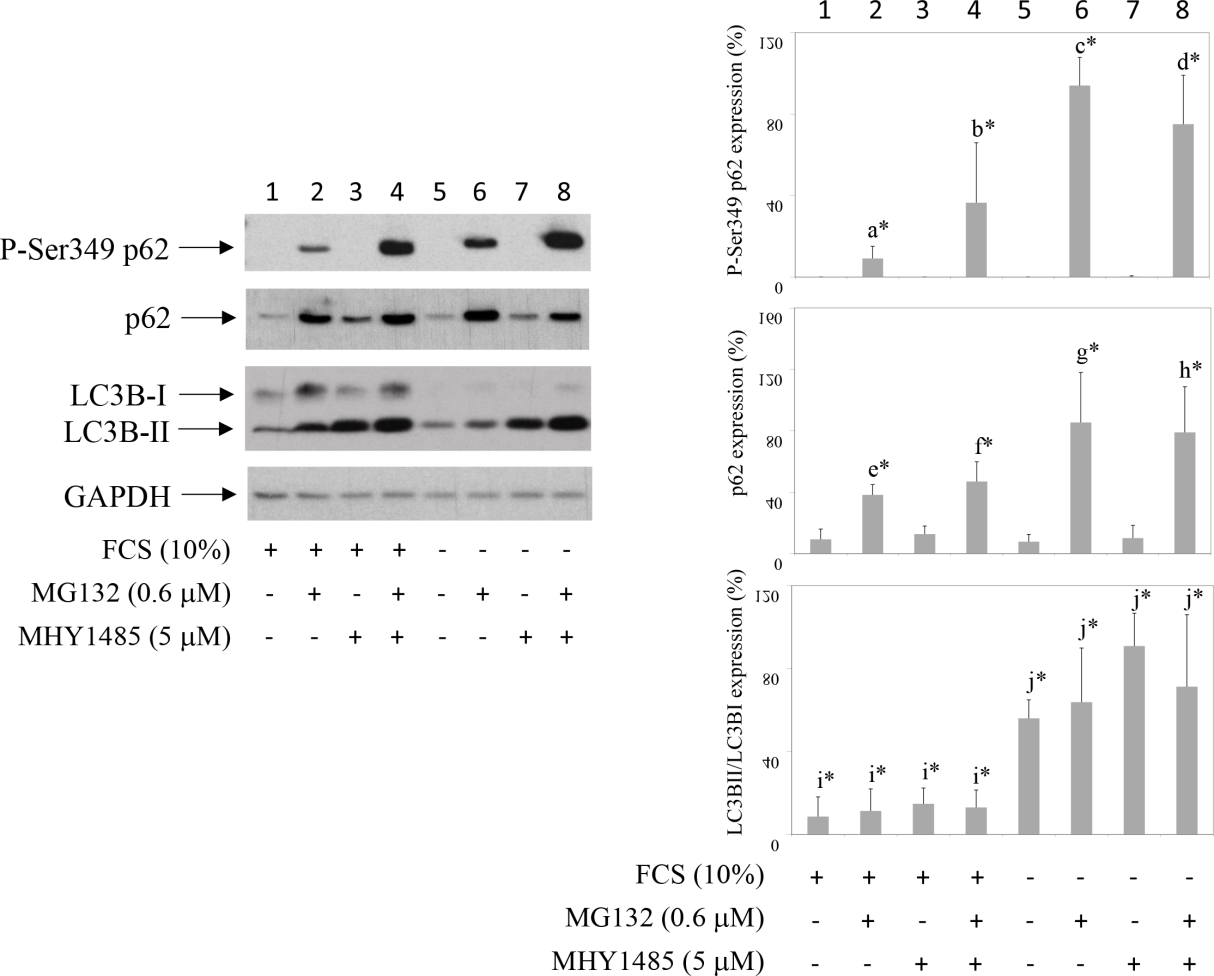
Inhibition of autophagy increases proteasome inhibitor-induced P-S349 p62 stabilization Human synovial fibroblasts were treated with proteasome inhibitor MG132 for 24 hours or concomitantly with MG132 and inhibitor of autophagy MHY 1485, in the presence or absence of 10% FCS. Western blots show P-S349 p62, p62, LC3B and GAPDH expressions in human synovial fibroblast extracts. Graphics represent average of protein expression from six different experiments done with synovial fibroblasts from six different OA patients. Values were calculated as in Figure 1. a^*^ is statistically lower from b^*^, c^*^ and d^*^; b^*^ is statistically lower from c^*^; e^*^ is statistically lower from f^*^, g^*^ and h^*^; f^*^ is statistically lower from g^*^; i^*^ is statistically lower from j^*^.

To test the effect of autophagy on P-Ser349 p62 degradation, cells were cultured in the presence of MG132 and/or autophagy inhibitor MHY1485. MHY1485 is an mTOR agonist and autophagy inhibitor that prevents fusion of autophagosome with lysosome [16]. Results showed that, in comparison to MG132 effect, significantly increased accumulation of P-Ser349 p62 as well as of p62 was detected when cells were exposed to concomitant MG132 and MHY1485 treatment (Figure 2, line 2 in comparison to line 4). Similar results were obtained if another autophagy inhibitor, NH_4_Cl, was used instead of MHY1485 (results not shown). However, statistical analysis of six different experiments showed that MHY1485 did not significantly increased P-Ser349 p62 in serum starved and MG132 treated cells (Figure 2, line 6 in comparison to line 8). These results showed that human synovial fibroblast P-S349 p62 and p62 are degraded by both proteasome and autophagy.

### MG132 protects whereas serum starvation sensitizes synovial fibroblasts to BAY 11-7085-induced cell death

**Figure 3:**
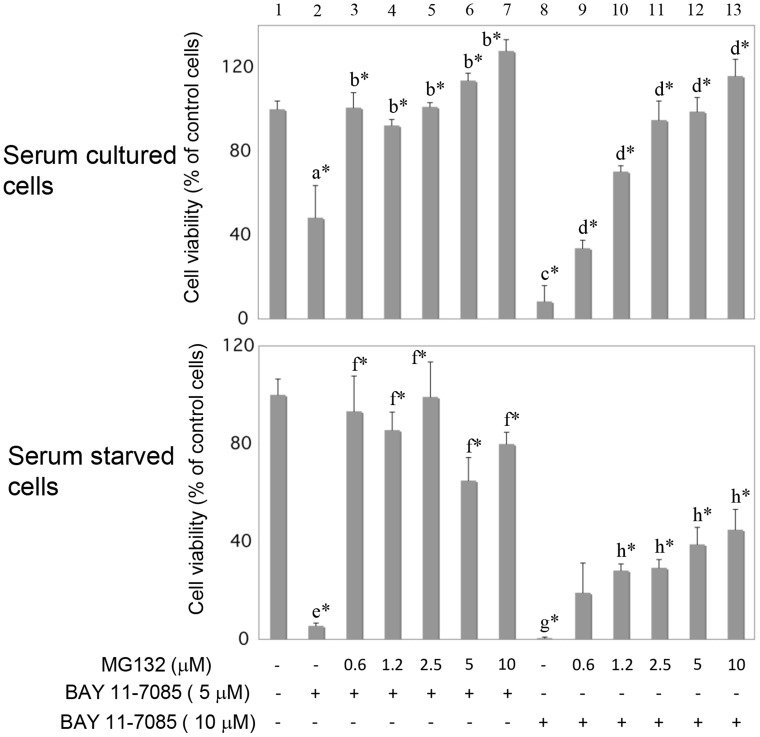
MG132 protects serum cultured and serum starved synovial fibroblasts from BAY 11-7085-induced cell death Human synovial fibroblasts were stimulated with indicated concentrations of MG132 for 24 hours, in the presence or absence of serum. BAY 11-7085 was then added for additional 2 hours. Cell survival, estimated by an MTS test, was expressed as a percentage of surviving cells compared with control cells. a^*^ is statistically lower than control cells and b^*^; c^*^ is statistically lower than d^*^ and a^*^; e^*^ is statistically different than control cells, f^*^ and a^*^; g^*^ is statistically lower than h^*^, e^*^ and c^*^.

Synovial fibroblasts were cultured in the presence or absence of MG132 (Figure 3) and then BAY 11-7085 was added for additional 2 hours. MTS test showed that MG132 markedly protected both serum cultured and serum starved cells from BAY 11-7085-induced cell death (Figure 3, line 2 in comparison to lines 3–7; line 8 in comparison to lines 9–13). However, serum starvation significantly sensitized synovial fibroblast to BAY 11-70085-induced cell death (Figure 3, lines 2 and 8 to be compared in serum cultured and serum starved cells, respectively). These results suggested that stabilization of P-S349 p62 and p62 may have pro-survival effects in synovial fibroblasts.

### Starvation induces RAD23B, a proteasome and NGLY1 interactive protein, and reversibly increases p62 molecular weight (MW). Starvation-induced higher MW p62 is mimicked by PNGase F

**Figure 4:**
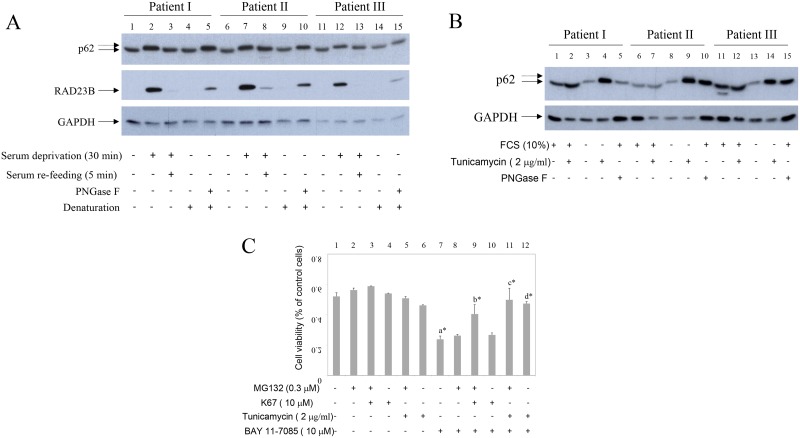
Starvation and PNGase F increase p62 MW and induce RAD23B Inhibitor of Keap1-P-Ser349 p62 interaction and tunicamycin have pro-survival effects on human synovial fibroblasts. (**A**) Serum cultured synovial fibroblasts from three different OA patients were starved for 30 minutes and then 10% FCS was added for additional 5 minutes. Western blots show p62, RAD23B and GAPDH expression in synovial fibroblast extracts. Control extracts were treated or not with PNGase F. Dash arrows show higher MW p62 form. (**B**) Serum cultured or serum deprived synovial fibroblasts from three different OA patients were cultured in the presence or absence of tunicamycin for 48 hours. Western blots show p62 and GAPDH expression in synovial fibroblast extracts. Control extracts were treated or not with PNGase F. Dash arrows show higher MW p62 form. (**C**) Human synovial fibroblasts were cultured in the absence or presence of MG132 (0.3 μM) and/or K67 inhibitor (10 μM) or tunicamycin (2 mg/ml) for 24 hours. BAY 11-7085 (10 μM) was then added for additional 2 hours. Cell survival, estimated by an MTS test, was expressed as a percentage of surviving cells compared with control cells.

We observed that, upon serum deprivation, p62 migrated slower on SDS gel (Figure 4A, lines 2, 7 and 12). Slower migrating p62 is shown by doted arrow. This p62 form was not recognized by P-S349 p62 antibody (Figure 1A, line 7 and Figure 2, line 5) and was not sensitive to λ protein phosphatase (data not shown). Concomitantly with increased p62 molecular weight (MW), serum deprivation also induced RAD23B, a proteasome and N-glycanase 1 (NGLY1) interactive protein [17, 18] (Figure 4A, lines 2, 7 and 12). In addition, both p62 increased MW and RAD23B expression were, within minutes, reversed with serum re-feeding (Figure 4A, lines 3, 8 and 13). Because NGLY1 is a human PNGase involved in endoplasmic-reticulum-associated protein degradation (ERAD) [19], these results suggested that de-glycosylation events maybe involved in p62 MW modification and/or p62 turnover. PNGase F treatment of denaturated synovial fibroblast extracts mimicked starvation-induced increase of p62 MW (Figure 4A, lines 5, 10 and 15). Of interest, p62 accumulated in synovial fibroblasts treated with tunicamycin, inhibitor of N-linked glycosylation (Figure 4B). In addition, tunicamycin significantly protected cells from BAY 11-7085-induced cell death (Figure 4C, line 7 compared to line 12). Furthermore, K67, inhibitor of both Keap1-P-S349 p62 interaction and Nrf2, that liberates Keap1 for its interaction with Nrf2 and stabilizes P-S349 p62 [10, 20], had synergistic effect with low, sub protective concentration of MG132 (Figure 4C, line 7 compared to line 9). These results showed that P-S349 p62, p62 and de-glycosylation events are positively involved in synovial fibroblast survival and that de-glycosylation events are involved in p62 turnover.

### Lentivirus mediated expression of phosphorylation-mimetic p62 mutant (S349E) markedly protects human synovial fibroblasts from BAY 11-7085-induced cell death

**Figure 5:**
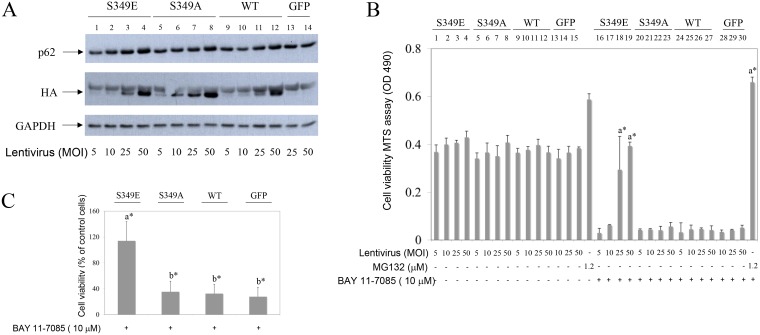
Phosphorylation-mimetic p62 mutant of Serine 349 (S349E), but not phosphorylation-defective mutant (S349A) or wild type (WT) p62, protects OA synovial fibroblasts from BAY 11 7085-induced cell death Synovial fibroblasts were transduced with lentivirus carrying HA tagged phosphorylation-mimetic (S349E) mutant, phosphorylation-defective (S349A) mutant, WT p62 or GFP control. After five days of infection BAY 11-7085 was added at indicated concentrations for additional 2 hours. Cell survival was determined by MTS test. (**A**) Western blots represent expression of p62, HA epitope and GAPDH in cell extracts of one representative experiment, after five days of infection, before BAY 11-7085 treatment. (**B**) Cell survival of the lentivirus transduced OA synovial fibroblasts shown in (A), after BAY 11-7085 treatment. Results of MTS test are shown as relative quantity of formazan in cell supernatant (OD490). a^*^ is statistically different from BAY 11-7085 treated control cells (expressing GFP). (**C**) % of cell survival compared to control cells (expressing GFP) and calculated as average values for three different OA patients transduced with lentivirus at 25 MOI. a^*^ is statistically different from b^*^.

**Figure 6:**
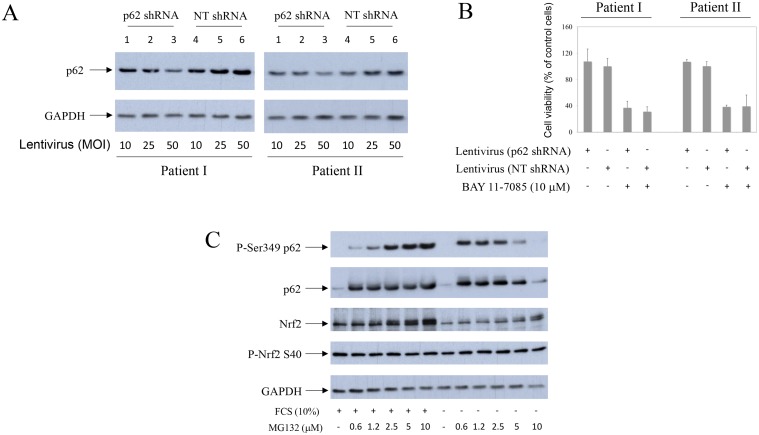
Partial knock-down of p62 does not change human synovial fibroblast fate upon BAY 11-7085 treatment Nrf2 is constitutively phosphorylated on Serine 40 in OA synovial fibroblasts. Synovial fibroblasts from two different OA patients were transduced with lentivirus carrying p62 shRNA or (non-targeting) NT shRNA as control, for five days. (**A**) Western blots show p62 and GAPDH expressions in cell extracts from two different OA patients. (**B**) After five days of lentvirus infection, BAY 11-7085 was added for additional 2 hours and MTS tests performed. Graphics show cell survival (% of control cells) of each donor cells, that were transduced with lentivirus carrying p62 shRNA or NT shRNA (50 MOI). (**C**) Synovial fibroblasts were cultured with different concentrations of MG132, in the presence or absence of serum, for 24 hours. Western blots show expression of P-S349 p62, p62, Nrf2, Nrf2 phosphorylated on Serine 40 and GAPDH in cell extracts of one representative OA patient. Experiments were repeated three times with cells from three different OA patients.

To test the direct effects of P-S349 p62 on synovial fibroblast survival, we have transduced cells with lentiviruses carrying either phosphorylation-mimetic p62-HA mutant (S349E), phosphorylation-defective p62-HA mutant (S349A), wild type p62-HA (WT) or control EGFP (GFP) (Figure 5A). Overexpression of phosphorylation-mimetic p62-HA mutant (S349E) markedly protected synovial fibroblasts from BAY 11-7085-induced cell death (Figure 5B, lines 18 and 19 in comparison with lines 29 and 30; Figure 5C), while phosphorylation-defective p62-HA mutant (S349A) and wild type p62-HA were without effect (Figure 5B, lines 22, 23 and 26,27, respectively; Figure 5C). Furthermore, a partial knockdown of p62, using lentivirus expressing p62 shRNA, did not change cell fate upon BAY 11-7085 treatment (Figure 6A, 6B). These results showed a prominent role of p62 phosphorylation on Serine 349 in human synovial fibroblast survival.

### OA human synovial fibroblasts constitutively express Nrf2 phosphorylated on Serine 40. MG132 increases Nrf2 expression

p62 phosphorylation on Serine 349 is known to activate Nfr2. We thus looked for Nrf2 and activated Nrf2 (*i.e.* phosphorylated on Serine 40) [21] in synovial fibroblasts that express P-S349 p62, upon MG132 treatment (Figure 6C). Results showed that while Nrf2 expression increased with MG132 concentration, phosphorylated Nrf2 was constitutively expressed in OA synovial fibroblasts (Figure 6C). These results suggested P-S349 p62 to be more involved in synovial fibroblast survival upon BAY 11-7085 treatment than Nfr2.

### Starvation-induced higher MW p62 is reversed by albumin to usual MW. PNGase F shifts p62 to faster migrating form in human synovial tissue extracts

**Figure 7:**
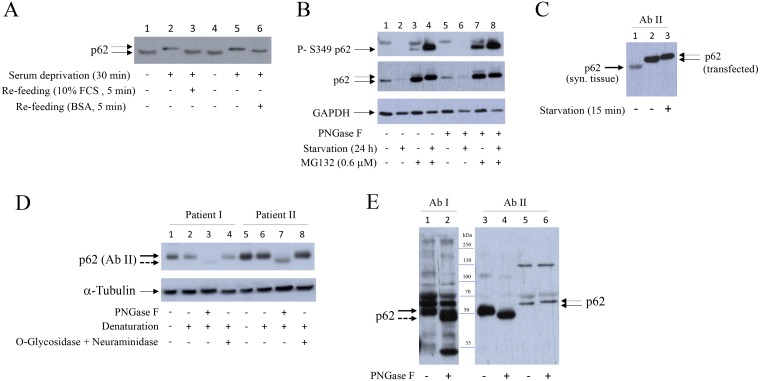
Bovine serum albumin reverses higher MW of p62 to usual MW PNGase F transforms p62 to de-glycosylation-like form with lower MW in human synovial tissue extracts. (**A**) Synovial fibroblasts were starved for 30 minutes and then 10% FCS or BSA (23 mg/ml) were added for additional 5 minutes. Western blot shows p62 expression detected with p62 (PW9860), Enzo Life Sciences. Dash arrow shows higher MW p62 form. (**B**) Synovial fibroblasts were cultured with or without serum, in the presence or absence of MG132, for 24 hours. Denaturated cell extracts were treated or not with PNGase F. Western show P-Ser349 p62, p62 (detected with p62/SQSTM1 (P0067), Sigma-Aldrich) and GAPDH expressions. (**C**) Western blot showing p62 detected in human synovial tissue and in serum cultured and serum starved HEK293 cells transfected with p62 [58]. p62 was detected by anti-SQSTM1/p62 (D5E2) (#8025), Cell Signaling. Dash arrow shows higher MW p62 form. Bold arrow shows p62 in synovial tissue. (**D**) Extracts of human synovial tissue were treated or not with PNGase F or with O-Glycosidase and Neuraminidase. Western blots show p62 and α-Tubulin expressions in human synovial tissue extracts of two OA patients. p62 was detected with anti-SQSTM1/p62 (D5E2) (#8025), Cell Signaling. Bold dashed arrow shows de-glycosylation like p62 form. (**E**) Comparison of p62 detection in extracts from human synovial tissue with p62/SQSTM1 (P0067), Sigma-Aldrich) (lines 1, 2) and anti-SQSTM1/p62 (D5E2) (#8025), Cell Signaling (lines 3, 4). Lines 5, 6: human synovial fibroblast extracts. All extracts were denaturated and treated or not with PNGase F. Dashed bold arrow shows de-glycosylation-like p62 form of smaller MW.

**Figure 8:**
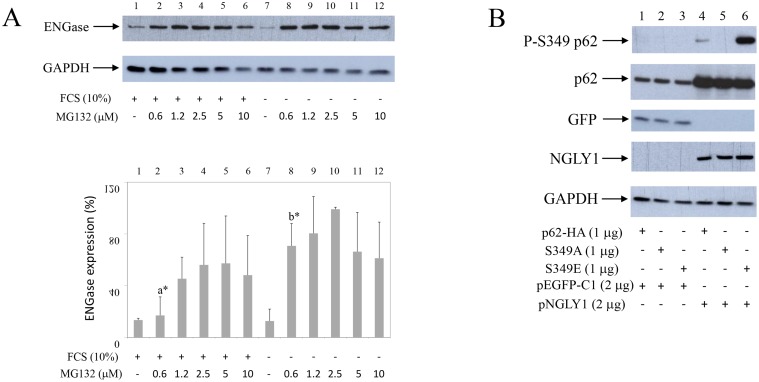
Starvation increases ENGase turnover in synovial fibroblasts Co-transfection of NGLY1 with p62 increases p62 expression in HEK293 cells. (**A**) Synovial fibroblasts were cultured with or without serum, in the presence of different concentrations of MG132 as indicated. Western blots show ENGase and GAPDH expressions in synovial fibroblast extracts. Graphics represent average of protein expression from three different experiments done with synovial fibroblasts from three different OA patients. Values were calculated as in Figure 1. a^*^ and b^*^ are significantly higher than controls. a^*^ is significantly lower from b^*^. (**B**) p62-HA or p62-HA mutant S349A that has no phosphorylation on Serine 349 or p62-HA mutant S349E that has constitutive mimetic phosphorylation on Serine 349 (8) are co-transfected with NGLY1 or pEGFP-C1 into HEK293 cells. Western blots show P-S349 p62, p62, GFP, NGLY1 and GAPDH expressions in HEK293 extracts.

We searched for serum constituent that is able to reverse starvation-induced higher MW p62 form. Results showed that higher MW p62 form, that appeared within minutes of starvation, was rapidly reversed by bovine serum albumin (BSA) (Figure 7A, line 6) or human serum albumin (results not shown). In the aim to further characterize the p62 MW modification we have treated extracts of synovial fibroblasts cultured in the presence of MG132 with PNGase F. PNGase F-induced shift of p62 (Figure 7B, line 5) also appeared in extracts of MG132 treated cells (Figure 7B, line 7). In synovial tissue extracts a p62 reacting band (marked by bold arrow) of smaller MW than transfected p62 was detected (Figure 7C, line 1 compared to line 2 and 3). This result suggested different p62 alternative splicing [22] in synovial tissue and synovial fibroblasts. Calculated MW of p62 is 47 kDa [23] and MW of spliced isoform is 38 kDa [22], but the p62 protein, which is thought to have complex covalent modifications, migrates at higher MW than 60 kDa [23]. PNGase F treatment of synovial tissue extracts produced, of interest, faster migrating band, suggesting de-glycosylation (Figure 7D, lines 3 and 7 and Figure 7E, lines 2 and 4). Specificity of commercially available p62 antibodies, probably due to different epitope specificity, was variable (Figure 7E and results not shown). In Figure 7E (lines 1–4), both of two different antibodies, used for the same synovial tissue extracts, revealed the presence of about 50 kDa form (Figure 7E, lines 1 and 3), marked with bold arrow, and PNGase F induced down-shifted form (Figure 7E, lines 2 and 4), marked with dashed bold arrow. However, several other p62 like forms are detected by the first commercial antibody while second commercial antibody detected only 50 kDa band and its PNGase F induced down-shifted form but had low specificity for p62 in synovial fibroblast extracts (Figure 7E, lines 5 and 6) and high sensibility for transfected p62 (Figure 7C, lines 2 and 3). In contrast, first commercial antibody had a strong affinity for p62 in synovial fibroblasts and it was used for the most experiments in this work (Figures 1, 2, 4, 5, 6, 7B, 8). These results suggested that p62 is N-glycosylated in synovial tissue. In line with this, sequence analysis showed two NXS N-glycosylation motifs [24] in human p62 protein: NWS at position 205–207 and NCS at position 330–332.

### MG132 stabilizes Endo-β-N-acetylglucosaminidase (ENGase) expression and starvation enhances ENGase turnover in synovial fibroblasts. NGLY co-transfection with p62 or p62 mutants S349A and S349E stabilizes p62 in HEK293 cells

Two human de-glycosylation enzymes have been cloned and characterized: NGLY1 [19] and ENGase [25–27]. To test possible effects of starvation on de-glycosylation events synovial fibroblasts were cultured in the presence or absence of serum and in the presence of different MG132 concentrations. Cell extracts were then tested by Western blot for human ENGase [25] (Figure 8A). Results showed that ENGase is expressed in synovial fibroblast (Figure 8A, line 1) and that MG132 stabilizes ENGase expression (Figure 8A, lines 2–6). MG132-induced stabilization of ENGase was significantly increased during starvation (Figure 8A, line 2 to be compared to line 8), suggesting increased turnover of ENGase during starvation-induced autophagy.

To test possible involvement of de-glycosylation on p62 and P-S349 p62 turnover, we have transfected human, HA epitope marked, p62 and its S349A and S349E p62 mutants [8], having no phosphorylation or constitutive mimetic phosphorylation on Serine 349, respectively, with human NGLY1 (pNGLY1) (Figure 8B). Results showed that pNGLY1 co-transfection, compared to control GFP clone (pEGFP-C1), markedly stabilized p62 and its mutants (Figure 8B, lines 1–3 compared to lines 4–6). Furthermore, pNGLY1 co-transfection also increased p62 phosphorylation on Serine 349 (Figure 8B, line 1 to be compared to line 4 and line 3 to be compared to line 6). These results strongly suggested that de-glycosylation enzymes are involved in p62 turnover.

## DISCUSSION

p62 has been reported to be degraded by autophagy: autophagy inhibitors increase p62 protein level [28] and p62 accumulates in cells of autophagy-deficient mice [29]. However, p62 also participates in UPS. It interacts with proteasome and depletion of p62 inhibits UPS [3]. Of interest, in neuronal cells p62 is ubiquitinated by Parkin on K13 and also degraded by proteasome [30]. MG132-induced proteasome inhibition results in p62 accumulation in human myelomonocyte cell line THP-1 [31]. p62 is phosphorylated on Serine 349 in Alzheimer disease patients [8]. Human SH-SY5Y neuroblastoma cells do not express P-Ser349 p62 but accumulates this phosphorylated form when stimulated with proteasome or autophagy inhibitors [8]. Our results presented here showed that, in human synovial fibroblasts, P-Ser349 p62 is degraded by both UPS and autophagy, that acted in synergy. We have observed previously that MG132 partially rescued PPAR γ from BAY 117985-induced degradation [32]. In this work, we have tested if MG132 also protects synovial fibroblasts from BAY 11-7085-induced cell death. Results showed that even nM concentrations of MG132 markedly protected synovial fibroblasts from BAY 11-7085-induced cell death. Protein analysis have shown that proteasome inhibitors led to P-Ser349 p62 accumulation that cannot be otherwise detected in synovial fibroblasts. Of interest, P-Ser349 p62 accumulation upon concomitant proteasome and autophagy inhibition markedly increased, and further increased during serum starvation. These results showed that p62 autophagic flux in synovial fibroblasts can be measured by P-Ser349 p62 accumulation, induced by concomitant cell treatments with nano molar concentrations of proteasome inhibitors and micro molar concentrations of inhibitors of autophagy. Since combination of proteasome and autophagic inhibitors often have undesirable toxic effects [33], the use of only nano molar concentrations of proteasome inhibitors might be also useful in cells other than human synovial fibroblasts. P-Ser349 p62 has been reported as accelerator of both protein aggregate formation and autophagic clearance [33]. Thus, in addition to p62 degradation [15], P-Ser349 p62 degradation may be also used as a marker of synovial fibroblast autophagy during starvation.

In this work, we have detected stabilization of RAD23B during serum starvation-induced autophagy. Rad23B is known for its roles in DNA repair and proteasomal degradation [34]. It binds both ubiquitin and proteasome, functioning as adaptor protein that itself escapes proteasomal degradation [35]. Of interest, Rad23 is involved in p53 turnover [36]. Coincidence of Rad23B up regulation during synovial fibroblast serum starvation, as well as its known interaction with NGLY1, suggest that RAD23B also may be involved in p62 turnover. Marked induction of RAD23B during serum deprivation of synovial fibroblast is a phenomena that will be further studied in another cell types including cancer cells, in which an decrease of RAD23B expression has been documented [37, 38].

Having interaction with NGLY1, RAD23B, apart from its function in DNA repair [39], is involved in degradation of misfolded glycoproteins in endoplasmic-reticulum [17, 40]. NGLY1 is one of de-N-glycosylating enzymes, whose activity is not linked to lysosome [41, 42], but it is involved in ERAD [26]. Very recently it was shown that NGLY1 activates cell protective Nrf1 [43]. Furthermore, NGLY1 inhibition inactivated Nrf1 and sensitized cells to proteasome inhibition [43]. Nrf1 and Nrf2 have both overlapping and distinct roles in antioxidant response [44, 45], respectively. S349T mutations was found in patient with Paget disease [12, 46]. This mutation is in the Keap1 binding region of p62. It has been shown that p62 mutated S349T is having less interaction with Keap1, less Nrf2 activation and thus links Paget disease to Keap1/Nrf2 [12, 47]. In the latest review p62 was proposed to be a switch between autophagy, apoptosis and cell survival [47]. Our results suggested that human p62 could be N-glycosylated because PNGase F converted synovial tissue extracts p62 into faster form, although this effect was not confirmed in isolated synovial fibroblasts. The reason for this discrepancy may lay in p62 alternative splicing [22]. Furthermore, PNGase F increased p62 molecular weight in isolated synovial fibroblasts. Similarly, not yet understandable increase of Npr1 molecular weight upon endo H treatment has been shown previously [48]. Two NXS motifs are present in human p62 protein: NWS at position 205–207 and NCS at position 330–332. These motifs represent potential N-glycosylation sites. These results suggested de-glycosylation events to be directly and/or indirectly, involved in p62 turnover. We thus overexpressed p62 with NGLY1 and found that NGLY1 markedly increases p62 expression in HEK293 cells, suggesting that NGLY1 is involved in p62 turnover. The future research is needed to distinguish if NGLY1 transfection have direct or indirect de-glycosylation effects on p62 or NGLY1 increases expression efficiency by anti-apoptotic effects [49]. Of interest, very recently, NGLY1 has been shown to de-glycosylate Nrf1 and change its turnover and function [43]. Furthermore, we have shown here that human ENGase [25, 27] turnover is significantly enhanced in starved human synovial fibroblasts. ENGase are distinct glycanase from NGLY1, that leaves N-acetylglucosamine attached to the deglycosylated protein. However, deletion of ENGase, can partially rescue lethality of NGLY1 deficient mice [27]. Further studies are needed to test how de-glycosylation events are involved in p62 fate.

Proteasome inhibitors can rescue cells from oxidative stress [50] as well as from apoptosis [9, 51]. In multiple myeloma UPS inhibitors are used as a treatment but many patients fail to respond due to p62 accumulation during proteasomal stress [52]. In our experimental model, we have detected p62 as cytoprotective protein during proteasome inhibition, but we cannot exclude Nrf2, mainly described as cytoprotective protein too [53]. We showed in this work that Nrf2 expression increased in MG132-treated OA human synovial fibroblasts, but that phosphorylated Nrf2 (Serine 40), an activated form of Nrf2 [21], was constitutively expressed in these cells. Also, very recently it was shown that BAY 11-7085 toxic effect in cancer cells include Nrf2 [54]. To distinguish p62 and Nrf2 effects, we have used K67, an Nrf2 inhibitor [20]. K67 inhibits Keap1-p-Ser349 p62 interaction and increases Nfr2 turnover [10, 20]. Our results showed that K67 had synergistic effect with low, sub protective concentration of MG132 to significantly protect synovial fibroblasts from BAY 11-7085-induced cell death. Furthermore, we showed here that lentivirus mediated overexpression of p62 S349E phosphorylation mimetic mutant significantly protect synovial fibroblasts from BAY 11-7085-induced cell death. In contrast, phosphorylation defective p62 S349A did not have protective effect but, of interest, wild type p62 neither. These results further confirmed important role of P-S349 p62 in OA synovial fibroblast survival. Similar results were obtained previously in human Huh7 and Hepa1 liver cell lines [10]. These cell lines are protected from Sorafenib- and Cisplatin-induced cell death when transduced by adenovirus carrying mouse phospho-mimetic p62 S351E (corresponding to human S349E) mutant, but not with mouse p62 defective phosphorylation p62 S351A mutant (corresponding to human S349A) or mouse wild type p62 [10].

Benefic effects of MG132 treatment has been shown in mice model of adjuvant arthritis [55] and mice model of collagen-induced arthritis [56]. Very recently, activation of p62-Keap1-Nrf2 axis was shown to suppress collagen-induced arthritis in mice through P-S349 p62 [13]. These results suggest that proteasome inhibitors may also activate p62-Keap1-Nrf2 axis *in vivo*. Indeed, proteasome inhibition in mice liver increase both autophagy and Keap1/Nrf2 pathway [57]. We showed in this work that proteasome inhibitor MG132 stabilized P-S349 p62 in synovial fibroblasts and that P-S349 p62 stabilization further increased during serum starvation as well as with concomitant inhibition of autophagy. Concomitant use of proteasome and autophagy inhibitors might act in synergy to stabilize P-S349 p62 *in vivo* too.

## MATERIALS AND METHODS

### Cell isolation and culturing

Human primary synovial fibroblasts from osteoarthritis (OA) patients were isolated and cultured as explained previously [14]. Serum cultured or serum deprived synovial fibroblasts were stimulated for 24 hours with proteasome inhibitor MG132 (CAS 133407-82-6), Calbiochem, and/or autophagy inhibitor, mTOR agonist MHY1485 (500554), or inhibitor of Keap1-P-Ser349 p62 interaction, K67 (SML 1922), Sigma. In some experiments an IkBα inhibitor BAY 11-7085 (Alexis Corp., San Diego, CA) was added for additional 2 hours. For autophagic flux determination [15], serum cultured or serum starved synovial fibroblasts were treated with Bafilomycin A1 (Sigma) for 24 hours.

### Western blotting

Human primary synovial fibroblasts and HEK293 cells were collected on ice in lysis buffer [32]. Human synovial tissue was cut, freezed and macerated in cold lysis buffer. Western blotting was done as explained previously [14, 32], by the use of following primary antibodies: p62/SQSTM1 (P0067), LC3B (L7543), GAPDH (G9545), α-Tubulin (T9026), Sigma-Aldrich, p62 (PW9860), Enzo Life Sciences, SQSTM1/p62 (D5E2) (#8025), RAD23B (D4W7F) (#13525), Cell Signaling, Phospho-p62 (SQSTM1) (Ser351) (M217-3), MBL, NGLY1 (A305-547A-T) Bethyl Laboratories, Inc., GFP (Y1030), UBPBio, ENGase (PA5-24531), Thermo Fisher Scientific, Nrf2 (MAB3925), R&D, and Nrf2 (Phospho S40) [EP1809Y] (ab76026), Abcam. Rabbit (#7074) and mouse (#7076) secondary antibodies were from Cell Signaling and mouse IgG2b (Star 134) from AbD Serotec.

### De-glycosylation tests

Cell and tissue extracts were denaturated and treated with PNGase F (P0704) or O-glycosidase (P0733) plus neuraminidase (P0720), New England BioLabs, for 1–3 h at 37° C, according to the manufacturer's instructions.

### Survival assay

Synovial fibroblasts were stimulated with different concentrations of proteasome inhibitor MG132 (CAS 133407-82-6), Calbiochem, for 24 hours and then BAY 11-7085 (Alexis Corp., San Diego, CA) was added for additional 2 hours. Survival assay, MTS (Promega, Madison, WI), was then performed for additional hour, according to the manufacturer's instructions. Cell survival was expressed as a percentage of surviving cells compared with control cells.

### Plasmids and DNA transfection

Human p62, cloned in pcDNA3.1 [58] was kindly provided by Dr Franco Venanzi, University of Camerino; HA tagged human p62, p62-HA, and its phosphorylation mutants Ser349A (phosphorylation defective) and Ser349E (phosphorylation mimetic) [8] were kindly provided by Dr Kunikazu Tanji, University of Hirosaki. Human NGLY1 cDNA, cloned in pCMV3 expression plasmid (HG15563-UT), was from Sino Biological Inc [59]. pEGFP-C1 was from Clontech. For transfection, HEK293 cells were seeded in 6-wells plates (BD Biosciences) (28 × 10^4^ cells/ 2 ml of culturing medium). Cells were transfected with 3 mg of DNA by the use of linear polyethyleneimine (MW 25,000) (Polysciences, Inc., Warrington, PA).

### Lentivirus transduction

For lentivirus transduction experiments, we created three gene transfer lentiviral plasmids by re-cloning of p62-HA, its phosphorylation mimetic mutant S349E and phosphorylation defective mutant S349A [8] as HindIII (blunted with T4 DNA polymerase)/XhoI fragment to EcoRV/XhoI sites of pLenti6/V5-D-TOPO vector (Invitrogen), that was previously modified by introduction of additional multiple cloning sites. EGFP cloned into pLenti6/V5-D-TOPO was used as control. Gene transfer lentiviral plasmid used for p62 (SQSTM1) knock-down experiments were pLV U6 shRNA hSQSTM1 #7235 (Hygro) (#180615-1046feu) (target sequence: CAGATGAGAAAGATCGCCTT) and non-targeting control pLV U6 shRNA NT (Hygro) (#VB180316-1189paa) (Vector Builder).

Lentivirus production was done by our GIGA Viral Vectors platform. Briefly, Lenti-X 293T cells (632180), Clontech, were co-transfected with gene transfer lentiviral plasmids, pSPAX2, Addgene and VSV-G encoding plasmid [60]. Viral supernatants were collected 48, 72 and 96 hours post transfection, filtrated (0.2 μM) and concentrated (100×) by ultracentrifugation. Titration was done with qPCR Lentivirus Titration (Titer) Kit (ABM).

For infection experiments human synovial fibroblasts were plated into 96-wells plates and day after inoculated with lentiviruses caring p62-HA, p62 (S349E), p62 (S349A), EGFP, shRNA p62 or non-targeting NT shRNA, at a multiplicity of infection (MOI) of 5–50. After 5 days of infection BAY 11-7085 was added for 2 hours and then MTS was performed. In parallel, synovial fibroblasts were plated into 24-wells plates and day after infected with lentiviruses caring p62-HA, p62 (S349E), p62 (S349A), EGFP, p62 shRNA or NT shRNA, at 5–50 MOI. After 5 days of infection cells were recuperated in lysis buffer [32] and analyzed by Western blot.

### Statistical analysis

*p* values were obtained using the Mann–Whitney test and Student's *t*-test. Values of *p* < 0.05 were considered as statistically significant.
